# SARS-CoV-2 detection by targeting four loci of viral genome using graphene oxide and gold nanoparticle DNA biosensor

**DOI:** 10.1038/s41598-022-23996-y

**Published:** 2022-11-12

**Authors:** Arman Amani Babadi, Shahrooz Rahmati, Rafieh Fakhlaei, Reza Heidari, Saeid Baradaran, Mostafa Akbariqomi, Shuang Wang, Gholamreza Tavoosidana, William Doherty, Kostya Ostrikov

**Affiliations:** 1 School of Energy and Power Engineering, Jiangsu University, Zhenjiang, 212013 Jiangsu China; 2 Department of Molecular Medicine, School of Advanced Technologies in Medicine, Tehran University of Medical Sciences, Tehran, 55469-14177 Iran; 3 School of Chemistry and Physics, Queensland University of Technology (QUT), Brisbane, 4000 Australia; 4 Centre for Agriculture and the Bioeconomy, Queensland University of Technology (QUT), Brisbane, 4000 Australia; 5 Centre for Materials Science, Queensland University of Technology (QUT), 2 George Street, Brisbane, 4000 Australia; 6 Centre for Biomedical Technologies, Queensland University of Technology (QUT), 2 George Street, Brisbane, 4000 Australia; 7 Food Safety and Food Integrity (FOSFI), Institute of Tropical Agriculture and Food Security, Universiti Putra Malaysia, 43400 Serdang, Selangor Malaysia; 8 Research Center for Cancer Screening and Epidemiology, AJA University of Medical Sciences, Tehran, 14117-18541 Iran; 9 New Technologies Research Center, Amirkabir University of Technology, Tehran, 15916-34311 Iran; 10 Applied Microbiology Research Center, Systems Biology and Poisonings Institute, Baqiyatallah University of Medical Sciences, Tehran, 14359-16471 Iran

**Keywords:** Diagnostic markers, Nanoparticles

## Abstract

The current COVID-19 pandemic outbreak poses a serious threat to public health, demonstrating the critical need for the development of effective and reproducible detection tests. Since the RT-qPCR primers are highly specific and can only be designed based on the known sequence, mutation sensitivity is its limitation. Moreover, the mutations in the severe acute respiratory syndrome β-coronavirus (SARS-CoV-2) genome led to new highly transmissible variants such as Delta and Omicron variants. In the case of mutation, RT-qPCR primers cannot recognize and attach to the target sequence. This research presents an accurate dual-platform DNA biosensor based on the colorimetric assay of gold nanoparticles and the surface-enhanced Raman scattering (SERS) technique. It simultaneously targets four different regions of the viral genome for detection of SARS-CoV-2 and its new variants prior to any sequencing. Hence, in the case of mutation in one of the target sequences, the other three probes could detect the SARS-CoV-2 genome. The method is based on visible biosensor color shift and a locally enhanced electromagnetic field and significantly amplified SERS signal due to the proximity of Sulfo-Cyanine 3 (Cy3) and AuNPs intensity peak at 1468 cm^-1^. The dual-platform DNA/GO/AuNP biosensor exhibits high sensitivity toward the viral genome with a LOD of 0.16 ng/µL. This is a safe point-of-care, naked-eye, equipment-free, and rapid (10 min) detection biosensor for diagnosing COVID-19 cases at home using a nasopharyngeal sample.

## Introduction

Coronavirus disease is a global public health issue with far-reaching effects on health systems and economies. On December 31, 2019, the first case of pneumonia of unknown origin was recorded in Wuhan, China. During the first week of January, the World Health Organization (WHO) announced these cases via social media^1^. The disease was recognized as a new type of severe acute respiratory syndrome β-coronavirus (SARS-CoV-2) in the same week, which impacted more than 200 countries^1,2^. The disease is extremely transmissible and has now become a pandemic on all continents^3^. As of July 2022, there have been more than 553 million confirmed cases of COVID-19 and over 6.3 million deaths globally, reported to WHO^4^. These data claim over 6% increase compared to the previous month (over 5.7 million new cases). The United States of America, India, Brazil, France, and Germany reported the highest number of laboratory-confirmed cases (more than 230 million cases in total) in August 2022^5^. The clinical COVID-19 patients can present a range from asymptomatic to mild, to severe and critical^6^. Vaccination programs, which are now underway with more than 200 COVID-19 vaccines, are expected to take months to complete, and even with success, countries may struggle to attain herd immunity^7,8^. Although vaccination is an important strategy in the fight against the COVID-19 pandemic^9^, many challenges, such as immune senescence and comorbidities in ageing populations, remain^10^. Despite vaccination's high efficacy, some infections after vaccination are to be expected, such as an outbreak associated with a SARS-CoV-2 R.1 lineage variant in a skilled nursing facility following a vaccination program in Kentucky, USA^11^. With the discovery of new strains of SARS-CoV-2 in different countries, they are considered 70% more dangerous than the existing COVID-19 virus^12^. The Delta variant (SARS-CoV-2 B.1.617), which was dominant in some regions of India and the UK, is one of the most horrifying examples of viral genome mutation. This variant was made due to three different mutations that occurred simultaneously in the N-terminal domain (NTD) and the receptor-binding domain (RBD) of the SARS-CoV-2 spike protein,^13^. These mutations increase the immune evasion potential of these variants and make them undetectable by the available reverse-transcriptase quantitative polymerase chain reaction (RT-qPCR) primers designed previously for SARS-CoV-2 detection. The RT-qPCR primers are extremely specific and sequence-dependent. It means that mutations in the target sequence (that may generate new SARS-CoV-2 variants of concern such as Omicron (B.1.1.529) variants and its newly dominant BA.4 and BA.5 subvariants^14^) prevent the formation of complementary structures and this set of primers cannot be used for the detection of most recent mutated variants anymore. The emerging BA.4 and BA.5 Omicron VOC are similar to earlier Omicron subvariants, but mutations in their spike proteins make it easier for them to penetrate human cells. They feature 39 mutations throughout the viral genome, which cause changes in the amino acid sequence of the spike protein^15^. These mutations can help Omicron subvariants escape antibodies created by past COVID-19 vaccines or prior infections^16^. However, the emerging VOC could reinfect naturally infected or vaccinated individuals^17^. Hence, researchers have focused on developing multiplexed RT-qPCR primers using plasmonic nanoparticles for point-of-care COVID-19 diagnosis^18^. In order to design a new set of RT-qPCR primers, the genomic sequence of mutated variants should be sequenced and studied to provide enough information, which is a time- and cost-consuming procedure. Thus, early, accurate, mutation insensitive, rapid, and capable of detecting new variants of COVID-19 is of great importance in the control of emerging pandemics and in decreasing the number of new cases^19–22^. Three main COVID-19 diagnostic methods, with their disadvantages and limitations, are (1) Chest CT Scan is limited to highly equipped hospitals and is not specific^23,24^, (2) Immunochromatographic is useless in early-stage screening and gives false-positive results^25^, and (3) RT-qPCR is dependent on a specific COVID-19 detection kit and is prone to generating false-positive results or negative cases, which leads to the spread of this infectious disease to a wider community^26^. RT-qPCR has the capability of real-time monitoring of amplified products, quick detection, and quantitation of infectious units, but raises technical difficulties for point-of-care downsizing compared with end-point polymerase chain reaction^18^. Besides, they are expensive, require costly instruments, substantial laboratory setup, and specialized skilled personnel for operation and data interpretation^27,28^. Therefore, a specific and ultrasensitive biosensor can be considered a promising solution to overcome the limitations of all the discussed methods by providing higher stability, specificity, sensitivity, accuracy in real-time detection, and continuous monitoring. In addition, biosensors are essential in remote diagnosis centres due to the inaccessibility of advanced diagnostic instruments. The biosensors can be distributed at the citizens' door and decrease the risk of spreading COVID-19 by skipping unnecessary trips to clinics for testing. Moreover, the fabricated biosensor in this study can be used in detecting other viruses and bacteria, by simply redesigning the DNA probes, based on the genetic information of a specific target of interest.

**Figure 1:**
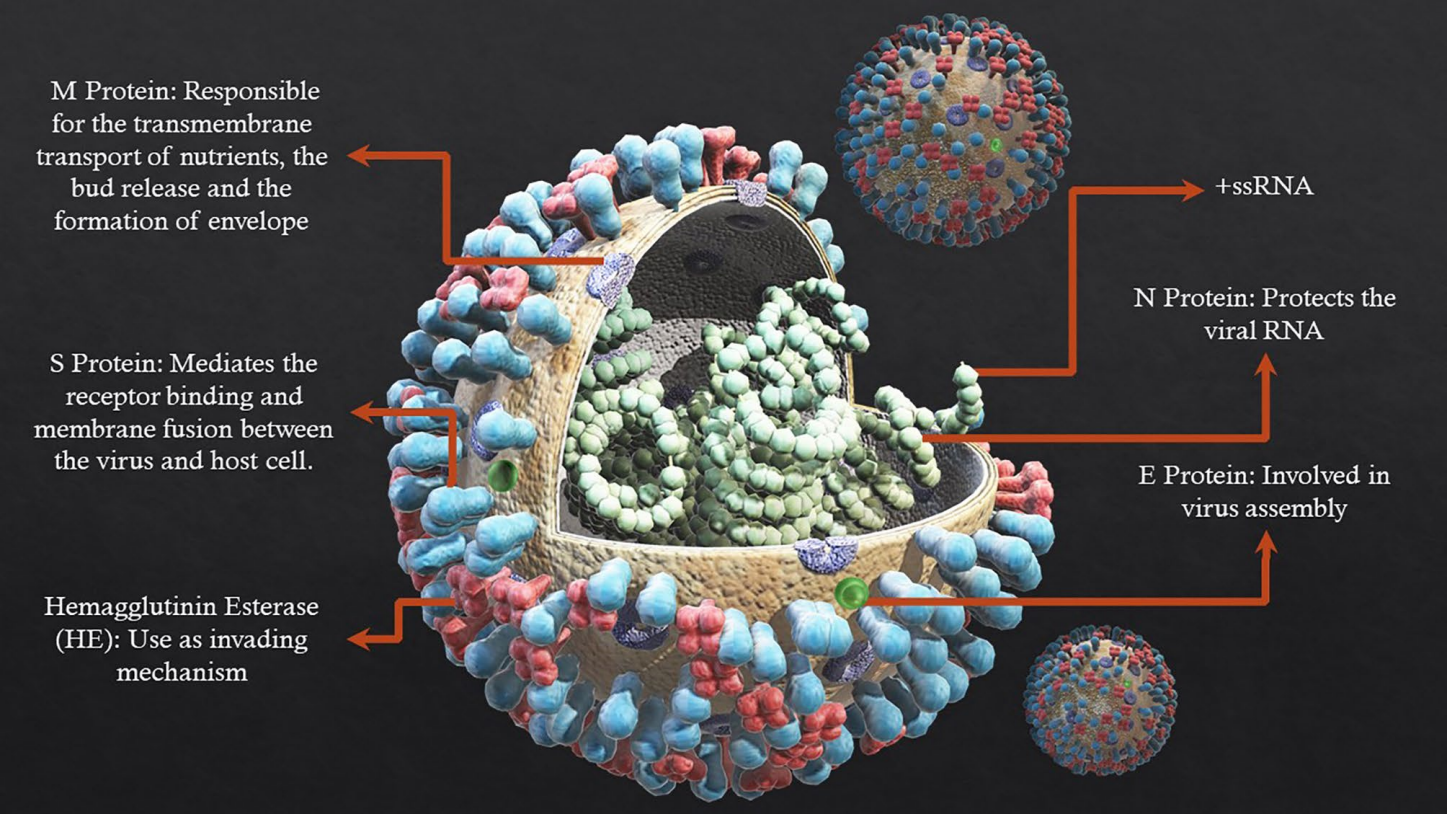
Schematic diagram of the SARS-CoV-2 structure and its several accessory proteins.

The SARS-CoV-2 provides us with three essential indicators or biomarkers^29,30^, which can be detected by biosensors, as illustrated in Fig. 1: antigen, antibody, RNA. Moreover, the structural proteins, including spike (S) glycoprotein, small envelope (E) protein, matrix (M) protein, and nucleocapsid (N) protein, and also several accessory proteins^31^ are illustrated in Fig. 1. The limiting factors that impact the DNA hybridization biosensor performance are the proper immobilization of the ssDNA probe and accessibility to target DNA^32–36^. Hence, a variety of nanoparticles were used in the fabrication process to enhance the immobilization and performance of biosensors owing to their high surface area^37,38^. One type of nanoparticle widely used in this process is gold nanoparticles (AuNPs), as it provides a suitable base for immobilization of the ssDNA probe through the latter 5’-phosphate anchors^39^. Furthermore, AuNPs are biocompatible^40^, have excellent conductivity^41^, are effective catalysts^42^ and have unique surface plasmon resonance^43^, high surface-to-volume ratio^44^, colorimetric-based biosensing capability^45^ and high surface to volume ratio^46,47^. All these unique properties make AuNPs suitable candidates for the fabrication of DNA hybridized biosensors. Moreover, simultaneous targeting of four different parts of the viral genome would eliminate the impact of new mutations in the virus genome on the biosensor function and reduce the mutation sensitivity of the fabricated biosensor. The developed biosensor can detect the infection prior to sequencing and study the genomic information of new variants. In this study, a highly sensitive and accurate DNA-based AuNPs biosensor was fabricated for the detection of SARS-CoV-2.

## Results

### Nanoparticles characterization

**Figure 2:**
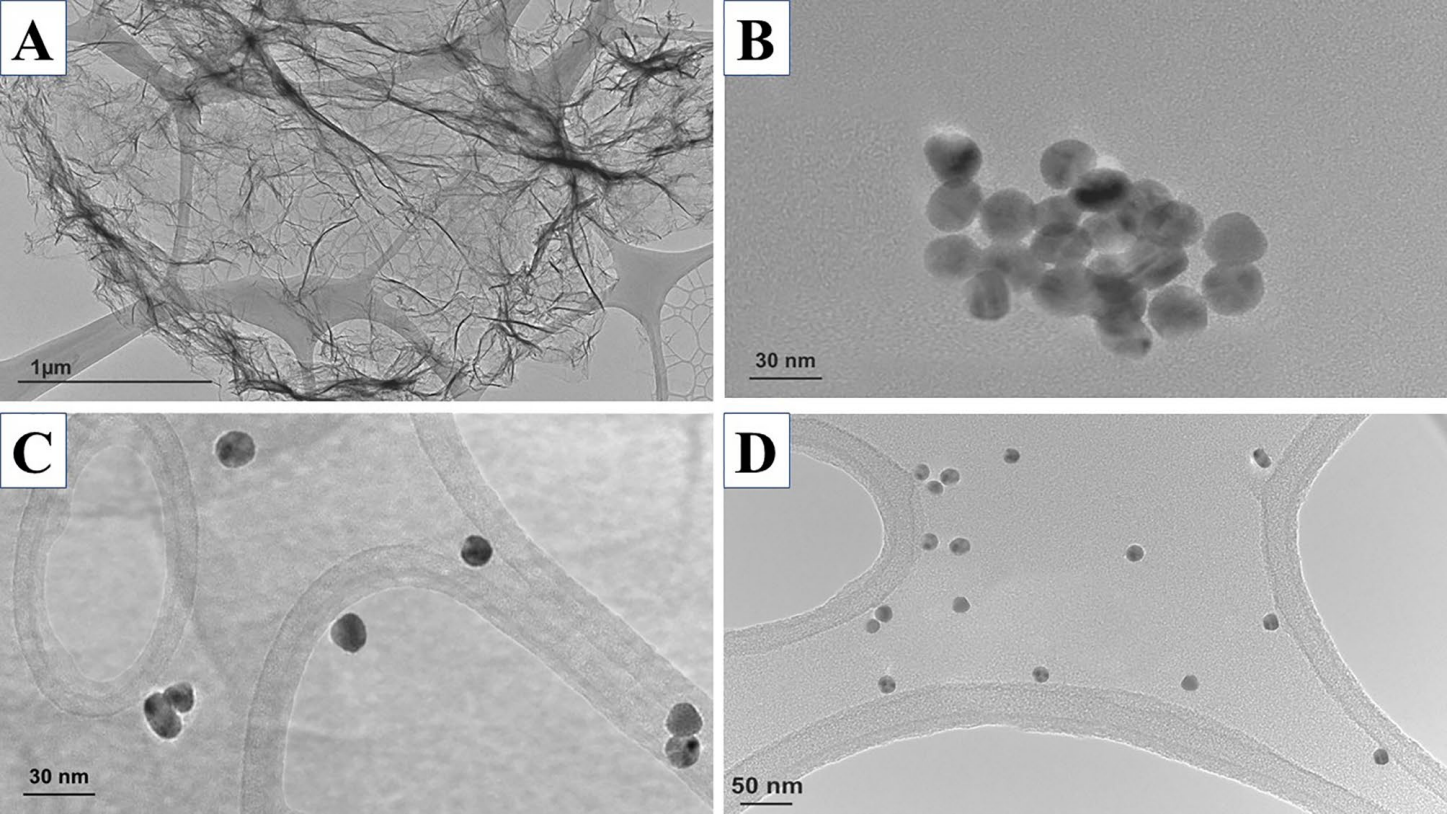
The HR-TEM micrograph of synthesized (**A**) GO, (**B**) AuNPs, (**C**) GO/AuNPs, and (**D**) SCVP1-3 capped AuNPs.

**Figure 3:**
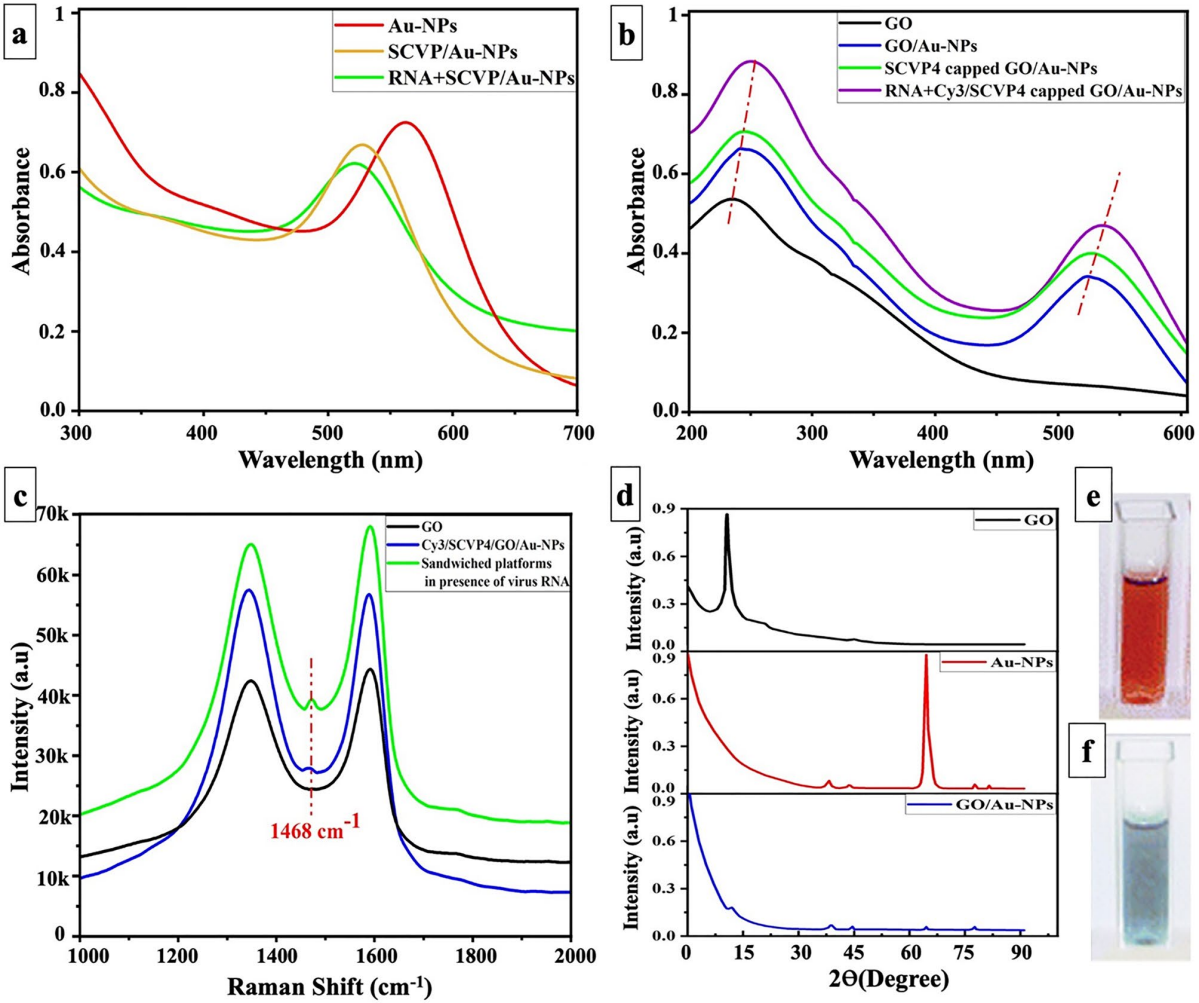
Characterization of synthesized nanoparticles and biosensor. (**a**) AuNPs, SCVPs capped AuNPs, virus RNA/SCVPs capped AuNPs UV–Vis absorption, (**b**) GO, GO/AuNPs, SCVP4 capped GO/AuNPs, virus RNA/SCVP4 capped GO/AuNPs UV–Vis absorption, (**c**) Raman spectroscopy of GO, Cy3/SCVP4 capped GO/AuNPs, sandwiched dual-platform in the presence of viral RNA, (**d**) XRD patterns of GO, AuNPs, and GO/AuNPs, Biosensor (**e**) before, and (**f**) 10 min after incubation with the SARS-CoV-2 RNA at 37 °C.

The HR-TEM micrograph of synthesized GO has presented in Fig. 2a. The HR-TEM micrograph shows that AuNPs are distributed individually without any visible aggregation (Fig. 2b). They all rang below 20 nm without the formation of any large particles. The HR-TEM micrograph of GO/AuNPs also reveals the successful hybridization of these nanoparticles (Fig. 2c). The synthesized AuNPs solution illustrates a clear ruby red color with the UV–Vis absorption band at 519 nm (Fig. 3a), as confirmed by Yuan, et al.^48^. The hybrid of GO/AuNPs provides us with two significant absorption peaks at 240 nm (the characteristic UV spectrum of GO^49,50^ at 232 nm) and 522 nm (the characteristic UV spectrum of AuNPs^51^) that justify the accomplished anchoring of AuNPs over the GO (Fig. 3b). The XRD analysis of Au-NP, GO, and GO/Au-NP has identified the crystalline material phases. The Au-NP XRD analysis shows five diffraction patterns at 38.25° (111), 44.4° (200), 64.68° (220), 77.69° (311), and 81.84° (222). The 38.18° (111) peak corresponds to the AuNPs’ predominant orientation. The XRD analysis of GO has a single peak at 10.65° since the graphite flakes were oxidized to GO. Finally, the XRD analysis of GO/Au-NP reveals peaks at 11.8° that contribute to GO, while 38.30° (111), 44.36° (200), 64.56° (220), and 77.60° (311) contribute to AuNPs (Fig. 3d). These peaks confirm the immobilization of AuNPs over GO. Besides, the XRD peak at 21.36° is related to the partial reduction of GO, which might be caused by ultrasonication of GO before characterization, as explained in other literature^52^.

### Capture probe capping of nanoparticles

The functionalization of AuNPs with the SCVPs cap shifted the visible color of AuNPs to red pinkish from the ruby red color of the gold nanoparticle solution (Fig. 3e). The UV–vis absorption of synthesized AuNPs shifted from 519 to 525 nm when they were capped with SCVPs (Fig. 3a). This visible naked-eye color shifting and UV–vis spectroscopy peak shifting are due to the AuNPs’ diameter increasing after capping with SCVPs and confirmed proper SCVPs capping^53^. In the same manner, the UV–vis spectroscopy of Cy3/SCVP4 capped GO/AuNPs was red-shifted to 524 nm from the 522 nm peak of GO/AuNPs and indicates the appropriate capping of thiolated Cy3/SCVP4 (Fig. 3b). However, Cy3/SCVP4 capped GO/AuNPs UV–vis absorption demonstrates a slight shift compared to SCVP capped AuNPs since the GO/AuNPs are much bigger and the changes in diameters are not significant. Besides, the HR-TEM micrograph of SCVP capped AuNPs indicates that the particles are individually dispersed without any visible aggregation or formation of a large entity in the absence of viral target RNA and prior to hybridization (Fig. 2d). In Fig. 2d, the mixture of successfully synthesized SCVP1-capped AuNPs, SCVP2-capped AuNPs, and SCVP3-capped AuNPs were presented, and there is no sign of agglomeration due to the interaction between AuNPs or SCVPs. There is also no complementary area among SCVPs, which eliminates the chance of unwanted agglomeration in the absence of target viral RNA.

Furthermore, Atomic Force Microscopy (AFM) topography (Fig. S1) confirms the well-dispersion of SCVP capped AuNPs, which, according to Csaki, et al.^54^ might be attributed to electrostatic repulsion. The AFM topography micrograph of SCVP4 capped GO/AuNPs further confirmed the AuNPs attachment on the GO sheet.

### Biosensor analytical performance

**Figure 4:**
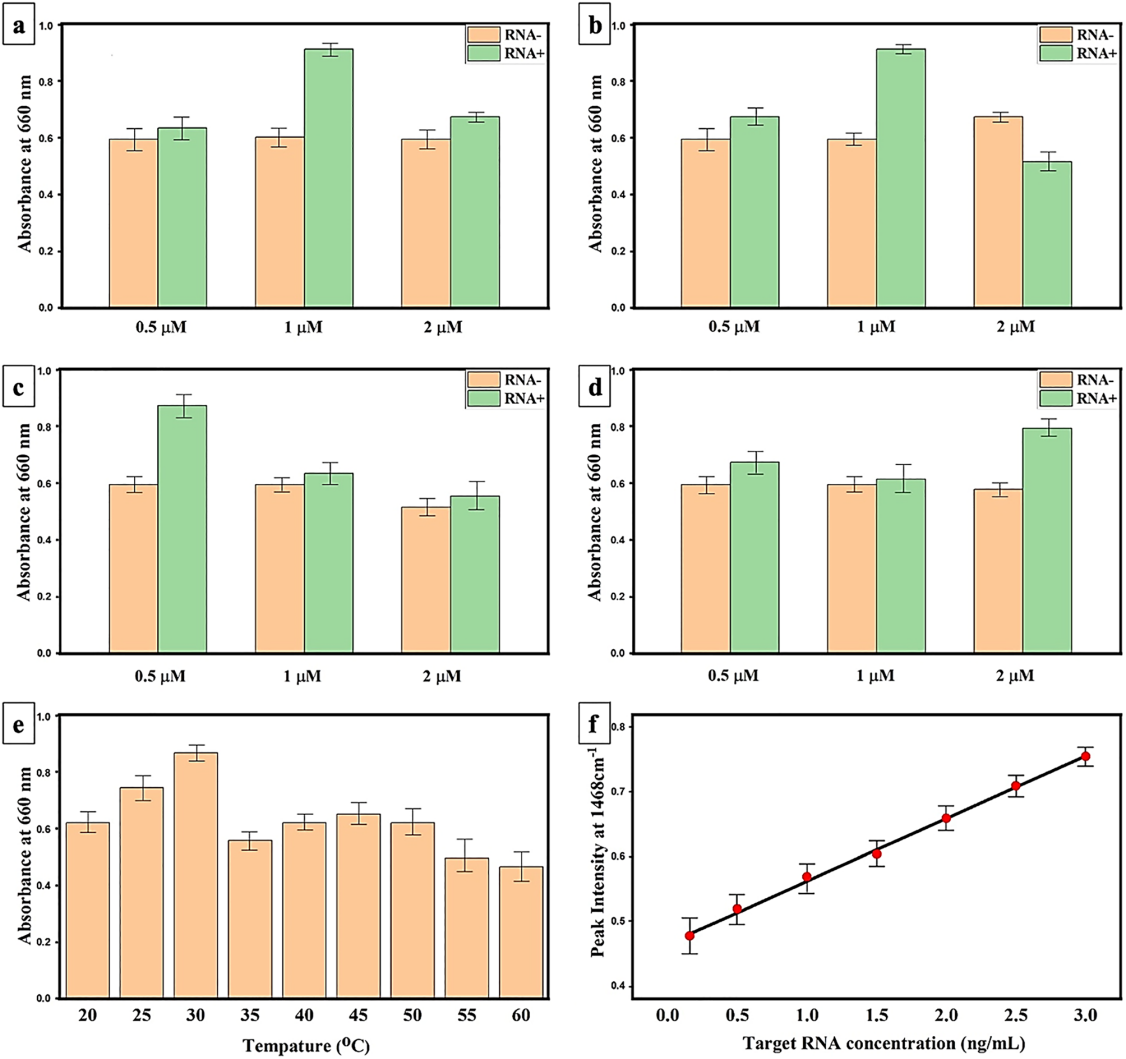
The analytic performance of fabricated biosensor. Absorbance at 660 nm for the AuNPs coated with 0.5, 0.1, and 2 µM of (**a**) SCVP1, (**b**) SCVP2, (**c**) SCVP3, and (**d**) SCVP4 upon the addition of SARS-CoV-2 RNA. (**e**) Effect of temperature on the response of SCVPM gold nanoparticles to the SARS-CoV-2 RNA when RNA concentration kept constant at 1 ng/μL. (**f**) The linear curve of SERS intensity at 1468 cm − 1 in response to target RNA concentration.

The sensitivity of SCVP-capped AuNPs towards the viral RNA was investigated by monitoring the UV–vis absorbance at 660 nm. To optimize and standardize the ratio of each SCVP to AuNPs, three different concentrations of SCVP1-3 (0.5, 1, and 2 µM) were used to functionalize the AuNPs. Moreover, the GO/AuNPs were capped with three different concentrations of SCVP4 (0.5, 1, and 2 µM). Among the 12 tested combinations, the 1 µM SCVP1 capped AuNPs (Fig. 4a), 1 µM SCVP2 capped AuNPs (Fig. 4b), 0.5 µM SCVP3 capped AuNPs (Fig. 4c), and 2 µM SCVP4 capped GO/AuNPs (Fig. 4d) provide the highest sensitivity towards the viral RNA (1 ng/µL) while incubating for 10 min. Therefore, the equivalent amounts of optimized SCVP1-3 capped AuNPs (1 µM SCVP1, 1 µM SCVP2, 0.5 µM SCVP3) and SCVP4 capped GO/AuNPs (2 µM SCVP4) were mixed and labelled as “SCVPM” to obtain the highest sensitivity of the biosensor toward the virus RNA. The introduction and agglomeration of SCVPM to viral RNA would cause a ~ 50 nm red-shift in the UV–vis absorbance at 660 nm. The effect of incubation temperature on the sensitivity of SCVPM was assessed. To this end, the concentration of 1 ng/µL viral RNA was incubated with SCVPM and their mixture in the range of 20–60 °C for 10 min (Fig. 4e). The optimum temperature was 35 °C. In addition, the change in 660 nm absorbance of SCVPM in response to various concentrations of viral RNA was studied to indicate the limit of detection (LOD). Logarithmic transformation of data is a useful and frequent method regarding the concentration versus intensity in SERS calibration curves. The log concentration vs intensity spreads out the data so that the shape and quality of the fit are clearly visible when the concentrations cover a wide range. When the data is exhibited at target RNA logarithmic concentration, the data yields a linear response with an R^2^ correlation coefficient value of 0.9832. The error bars were calculated by three repeated measurements of each concentration of target SARS-CoV-2 RNA. The LOD was designated to be 0.16 ng/µL for SCVPM (Fig. 4f). The 10 Kb distance between the probe-target location of SCVP4 and SCVPM in SARS-CoV-2 RNA prevents any interference of probe hybridization with virus RNA while the color shifting due to surface plasmon resonance remains unpretentious (Fig. 4b). The linear response and continuous increase in absorbance justify the reliable and constant response of the biosensor toward viral RNA.

### Detection of SARS-CoV-2

The fabricated biosensor is a dual platform sandwich DNA biosensor for the detection of SARS-CoV-2 to increase accuracy, selectivity, and sensitivity. The first developed platform contains SCVP1-3 capped AuNPs, while the second platform employs Cy3/SCVP4 capped GO/AuNPs to detect viral RNA. Hence, the dual-platform detection steps are as follows:

#### Naked eye detection

The first platform of this biosensor has been designed to report the presence and detection of SARS-CoV-2 RNA in the visual appearance noticeable to the naked eye. This would make the test results approachable even by the layman. As previously stated, the introduction of viral RNA to the first platform of the biosensor (SCVP1-3 capped AuNPs) at 35 °C for 10 min would result in an agglomeration between these two, which would increase the 660 nm absorbance by ~ 50 nm and induce the solution color shift from red to blue (Fig. 3f), which is in accordance with the report of previous studies^31,55^. This is a significant and distinguishable marker in the visual appearance of the test solution which can be observed by the human naked eye. The intensity of the blue color correlates with the concentration of viral RNA. To eliminate false-positive results, the second platform of biosensor has been designed and employed.

#### Fluorometric detection

Since there might be the possibility of AuNP self-agglomeration in the absence of viral RNA due to available ions and metabolites in the test sample, this biosensor employed Cy3/SCVP4 capped GO/AuNPs as a second transducer platform. In the case of positive results, the second platform could act as a Surface-enhanced Raman scattering (SERS) owing to its fluorescence quenching and narrow spectral bandwidth curve for the intensity of the Cy3 peak at 1468 cm^-1^ (Fig. 3c). The coupling of the SCVP1-3 capped AuNPs platform and the Cy3/SCVP4 capped GO/AuNPs platform for target viral RNA generates a locally enhanced electromagnetic field ‘hot spot’ and significantly amplifies the SERS signal. The hybridization of SCVP4 with virus RNA leads to the proximity of Cy3 and AuNPs that induce strong SERS signals. The SERS intensity is enhanced by both the local electromagnetic field due to the nanoparticle aggregated hot spots originating between the GO-AuNP and AuNPs and the chemical enhancement between the AuNPs and the Cy3. The SERS signals confirm the presence of the target DNA sequence in the fabricated dual-platform biosensor. This design suggests a novel, highly accurate, and selective biosensor for both naked-eye detection and SERS-based detection of SARS-CoV-2 in biospecimens.

## Discussion

In this study, we report the design and fabrication of a dual-platform DNA/GO/AuNP biosensor. This biosensor has been developed based on the antisense-specific binding ability of oligonucleotides toward viral RNA and the unique surface plasmon resonance of gold nanoparticles. To this end, four oligonucleotide probes were designed and immobilized on two different nanomaterial platforms. These four oligonucleotide probes targeted different regions of the virus genome (N-gene, E-gene, and ORF1ab) at the same time and increased the selectivity and sensitivity of the biosensor. The first three probes were modified by the thiol group to be immobilized on synthesized AuNPs to provide the first platform. The second platform was fabricated by immobilization of the fourth thiol-modified probe containing the Cy3 marker on the GO/AuNPs hybrid. The optimized and standardized first platform detects SARS-CoV-2 positive cases within 10 min after the addition of total extracted RNA from the nasopharyngeal sample. The efficient and selective agglomeration of SCVP-capped AuNPs in the presence of viral RNA would significantly influence the surface plasmon resonance of AuNPs with a 50 nm red-shift in 660 nm UV–vis absorbance. This leads to shifts in the color of the solution from red to blue, which can be recognized by the human naked eye. To eliminate the possibility of false-positive results, the second platform was designed based on SERS. Hybridization of two platforms together with the target virus RNA would lead to the formation of a sandwich configuration. This conformation enhances the fluorescence quenching of Cy3 (RAMAN tag), confirming the positive result and the existence of viral RNA in the sample with a LOD of 0.16 ng/µL. Since the fabricated biosensor targets four different regions of SARS-CoV-2 viral genome, in case of mutation in one of the target sequences, the other three probes could detect the new variants prior to any sequencing. Moreover, visible biosensor color shifting is another benefit of fabricated biosensor which eliminates the dependency on trained personnel and facilitates the detection process. This equipment-free and safe point-of-care biosensor rapidly (10 min) detect COVID-19 using a nasopharyngeal sample. According to Table 1, this LOD is much better than some of the newly developed metal-based biosensors. Furthermore, the employed methodology in this study ensures its feasibility even with a new mutation in the virus genome during its global spread since four different regions of viral RNA have been targeted simultaneously. Moreover, this biosensor has been validated for real-time sample analysis extracted from confirmed COVID-19 positive patients. As a result, this study reports the fabrication of sensitive, specific, user-friendly, rapid, and sophisticated equipment-free biosensors, which can be utilized directly by layman end-users and are potentially appropriate for future diagnosis of other viruses and pathogens with proper adjustments.

**Table 1:** Comparison of currently fabricated biosensor for detection of SARS-CoV-2.

No	Detection strategy	Nanomaterial	LOD (ng/µL)	Target	References
1	Entropy-Driven Amplified Electrochemiluminescence (ECL)	Ru (bpy)_3_^2+^	2.67	RdRp gene	^56^
2	Platinum/Titanium Electrodes on The Glass Substrate	Ti/Pt	0.843	RdRp gene	^57^
3	Electrochemiluminescence (ECL) immunoassay	Au-g-C_3_N_4_	4.37	RdRp gene	^58^
4	Electrochemical Biosensor	Gold nano-needle	0.68	S and Orf1ab genes	^59^
5	Colorimetric and electrochemical	AuNPs	0.48	Spike Monoclonal Antibody	^60^
6	Colorimetric SERS-based biosensor	GO/AuNPs	0.16	RdRp, E, and N gene	This study

It is believed that the designed colorimetric and SERS bioassays could be considered for the detection of various pathogens (e.g., Haemophilus influenzae, SARS-CoV, MERS-CoV, Ebola virus, Zika virus, and dengue virus) by modifying their oligonucleotide probe sequences according to the genome sequence of the target pathogens.

## Materials and methods

### Chemicals and instruments

All the chemicals used in this study were procured from reputable commercial suppliers without any further purification. All selected oligonucleotides were synthesized and provided by Integrated DNA Technologies (IDT), Singapore. Thermo Scientific GenPure UF/UV, Ultrapure Water System was used to prepare the ultra-pure water (UPW) for this study. The nasopharyngeal specimens were collected from the positive confirmed cases of COVID-19 inpatients (double-checked with two separate RT-qPCR tests) from Baqiyatallah University of Medical Sciences and stored at 4 °C for same-day analysis at the School of Advanced Technologies in Medicine, Tehran University of Medical Sciences, Tehran, Iran. Accordingly, informed consent from all subjects and/or their legal guardian(s) for study participation was obtained. This study was reviewed and approved by the ethical committee of the Baqiyatallah University of Medical Sciences, Iran (IR.BMSU.REC.1399.183) and all the experiments were performed in accordance with the relevant guidelines and regulations.

### Biosensor development strategy and principle

**Figure 5:**
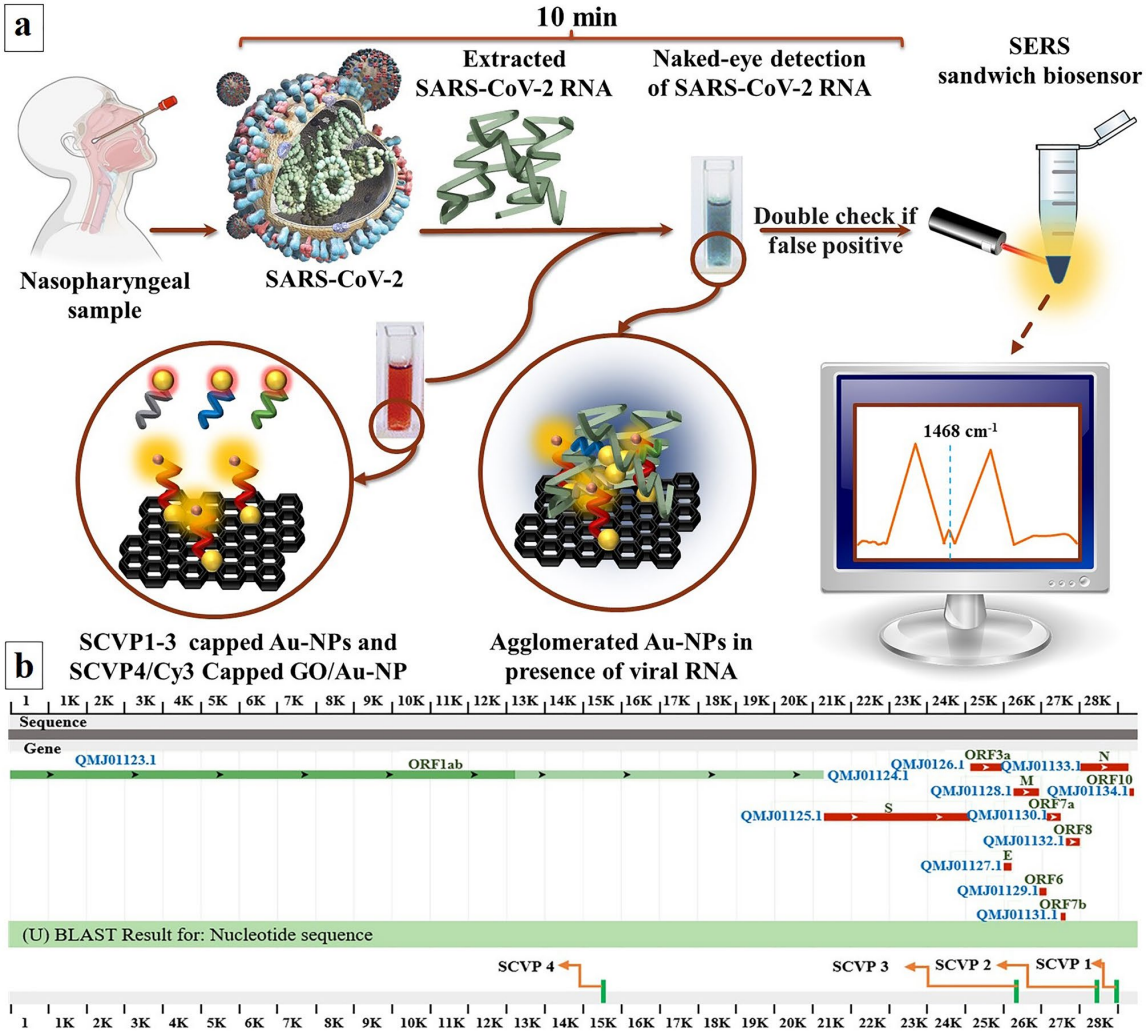
(**a**) The schematic illustration of the dual-platform biosensor and detection mechanism of dual-platform biosensor and (**b**) Schematic illustration of SARS-CoV-2 RNA and designed SCVP complementary regions.

Researchers who studied the genomic sequence of SARS-CoV-2 in the current pandemic have reported the continuous mutations inside the virus genome, which makes it challenging to design a single specific diagnostic probe. According to the Centers for Disease Control and Prevention (CDC) and WHO up to this date, four different major global variants of concern (VOC), were labeled in 2021 as alpha, beta, gamma, and delta^61,62^. These variants were generated due to the mutations accrued in different regions of the virus genome, as presented in Table 2. As it can be extracted from Table 2, these VOCs were caused by a simultaneous mutation in different parts of the virus genome. Unfortunately, current detection methods such as RT-qPCR only target one specific locus of the virus genome, which would be useless if a mutation occurred at those specific loci. Hence, in this study, we have focused on three genomic regions, which are more conserved: (1) RNA-dependent RNA-Polymerase gene (RdRp), (2) Envelope protein gene (E gene), and (3) Nucleocapsid phosphoprotein gene (N gene). To increase the accuracy of the biosensor, eliminate the effect of new, unknown mutations, and decrease false (positive/negative) results, four different SCVPs complementary to different regions inside these conserved areas were designed. Although three of these SCVPs were capped on the AuNPs for visual naked-eye detection of the viral genome, one of them was additionally labelled with Cy3 fluorescent dye and capped over the GO/Au-NP. The Cy3/SCVP4 capped GO/AuNPs is an extra diagnosis platform to increase the accuracy of the biosensor for quantitative purposes. The schematic view and detection mechanism of the dual-platform biosensor is illustrated in Fig. 5a.

**Table 2:** Major global SARS-CoV-2 variants of concern^63–66^.

WHO Lable	Designated VOC	Notable mutation	Transmissibility	Mortality
Alpha	December 2020	E484K, 69–70del, N501Y, P681H	+ 30%	+ 59%
Beta	January 2021	K417N, E484K, N501Y	+ 25%	Possibly increased
Gama	January 2021	K417T, E484K, N501Y	+ 38%	+ 50%
Delta	May 2021	L452R, T478K, P681R	+ 97%	+ 137%

### Antisense oligonucleotide design

The whole genomic data sequence of the virus has been obtained from NCBI, the WHO, and GISAID (Munich, Germany). By following the WHO guideline, studying the mutation-prone area and mutation rate, and by employing the Basic Local Alignment Search Tool (BLAST) (https://blast.ncbi.nlm.nih.gov/Blast.cgi), four oligonucleotide sequences were designed for the present study, which were labelled as SARS-CoV-2 Probes (SCVP) in Table 3. Furthermore, the SCVPs were verified by *in-silico* studies such as multiple sequence alignments with the other potential non-target genomes using MEGA5 software (http://www.megasoftware.net/) and BLAST (Tables S1-4) as presented in Fig. 5b. The physical characterization of the designed SCVPs is listed in Table S5. SCVP1 and SCVP3 were functionalized by the thiol modifier at the 5’ end, while SCVP2 and SCVP4 were thiolated at the 3’ end. Furthermore, SCVP4 has the Cy3 labelling at the 5’ end. Besides, the 6-carbon spacer has been placed in between the DNA bases and the thiol group to eliminate the interference effect of AuNPs in RNA/DNA hybridization. These functionalized SCVPs are capped to prevent gold nanoparticle agglomeration in the presence of SARS-CoV-2 RNA due to their complementary binding properties. The selected target sequences in SARS-CoV-2 RNA and their complementary DNA employed in the fabrication of the SCVPs are listed in Table 3.

**Table 3:** Selected target sequences and their complementary DNA (SCVPs).

SCVP	Probe sequence ($5^{^{\prime}} \to 3^{^{\prime}}$)	Target sequence ($5^{^{\prime}} \to 3^{^{\prime}}$)
1	GGCCAATGTTTGTAATCAGT	ACTGATTACAAACATTGGCC
2	ATTGTTAGCAGGATTGCGGG	CCCGCAATCCTGCTAACAAT
3	CGAAGCGCAGTAAGGATGGCTAGTG	ACACTAGCCATCCTTACTGCGCTTCG
4	GCATCTCCTGATGAGGTTCCACCTG	CAGGTGGAACCTCATCAGGAGATGC

### Synthesis of nanoparticles

Graphene oxide (GO) was synthesized through the modified Hummer’s method^67^. A mixture of 10:1:0.5 of concentrated H_2_SO_4_/H_3_PO_4_/KMnO_4_ was added to 1 g of graphite flakes and stirred for 3 days at 40 °C while the mixture’s color shifted from dark purple-green to dark brown. To terminate the reaction, 150 g of ice cubes and 7 ml of H_2_O_2_ were added to the solution. The yellowish graphene oxide was washed with HCl, rinsed with deionized water, and centrifuged three times until a pH of 5 was achieved. The GO pellets were collected, freeze-dried for 36 h, and ground into powder. The gold nanoparticles were synthesized following the Wang, et al.^68^ method. A total of 35 mL of sodium citrate solution (40 mM) was added to the preheated (90 °C) 350 mL of HAuCl_4_·3H_2_O (1 mM) while staring on top of the hotplate, which turned the mixture’s color dark red. The solution refluxed for at least 20 min and cooled down to room temperature before passing through the 0.45 µm syringe filter. The synthesized AuNPs were stored in a dark bottle at 4 °C. Finally, the hybridization and synthesis of GO/AuNPs. Although there is plenty of literature published on the modification of GO with AuNPs^69–71^, the Zhu, et al.^72^ technique is a reliable method that fits our biosensor application in this study has been selected. A 10% (w/v) aqueous solution of synthesized GO with ultra-pure water was ultrasonicated for 2 h before the addition of 0.1 L of HAuCl_4_.·3H_2_O (1 mM). This suspension was stirred for another hour to encourage the interaction of the Au^+^ ion with GO. The aqueous solution was heated up to 90 °C, and then 2 mL of sodium citrate solution (300 mmol) was added, and the stirring continued for the next 4 h. After cooling down the solution to room temperature, it was centrifuged at 6000 rpm for 2 h followed by a multiple step washing with ultra-pure water to eliminate the unbounded AuNPs. The synthesized GO/AuNPs hybrid was resuspended in ultra-pure water and stored in dark bottles inside the fridge for future characterization and applications.

### Nanoparticle functionalization

The thiolated SCVP oligonucleotides require chemical activation before the functionalization of AuNPs. To this end, 10 mM Tris-(2-Carboxyethyl) phosphine hydrochloride (TCEP), a reducing agent, was prepared in a fresh batch and added to 4 separate 2 mL microtubes containing 100 µL of each SCVP. These 4 tubes were incubated at 25 °C for 1 h to promote the reduction of disulfide bonds. The activated thiol SCVP1-3 was added to 3 separate microtubes containing synthesized AuNPs (400 µL) while the activated thiol SCVP4 was added to the synthesized GO/AuNP hybrid microtubes. All the microtubes were covered in aluminium foil to prevent light, vortexed, and incubated at 25 °C for 18 h. Afterwards, 500 µL PBS buffer (10 mM, pH 7.4) and 100 µL Tween 20 (0.1%) were added to each microtube and incubated for another 30 min. The next step is DNA functionalized nanoparticle salt-ageing which 10 µL NaCl (1 mM) increment was added during 1 h to each SCVP tube in the step of 3 min. This gradual addition of NaCl changes the parallel conformation of oligonucleotides to an upright conformation and facilitates the RNA/DNA hybridization process. The SCVP microtubes were incubated in a dark environment at 25 °C for 48 h. The 4 DNA functionalized nanoparticles were centrifuged at 8000 rpm for 20 min, the supernatant that contains unbounded free DNA was disposed of, and nanoparticle pellets were dispersed in PBS:NaCl buffer (1:100). This washing process was repeated 3 times, and the final nanoparticle pellets were dispersed again in PBS:NaCl buffer (1:100) and stored separately at 4 °C.

The SCVPs will hybridize with their target sequence in the viral genome while SCVPs1-3 cap the AuNPs. This would lead to the agglomeration of AuNPs and a change in the visual appearance of the color of the solution. AuNPs are well-known for their photophysical properties, triggered by influencing nanoparticle size and shape on surface plasmon resonance (SPR). Therefore, the aggregation of AuNPs results in a color shift of the solution from red to blue, owing to interparticle SPR coupling. On the other hand, in some cases, there might be false solution color changes (false-positive result). Since the GO/AuNPs were capped with the SCVP4 containing Cy3 as a RAMAN tag, this false positive result can be assessed by the fluorescence quenching property of Cy3 (surface-enhanced Raman scattering). The intensity peak at 1468 cm^-1^ correlates with Cy3 and AuNP quenching properties and confirms the presence of the viral genome^52,73^. In the case of peak absence at 1468 cm^-1^, the test would be considered a false positive.

### Ethical approval

This study was reviewed and approved by the ethical committee (IR.BMSU.REC.1399.183) of the Baqiyatallah University of Medical Sciences, Tehran, Iran.

## Supplementary Information

Supplementary Information.

## Data Availability

All data are available in the main text or supplementary materials.
