# Supplementary material for: SARS-CoV-2 detection by targeting four loci of viral genome using graphene oxide and gold nanoparticle DNA biosensor
Source: Sci Rep. 2022 Nov 12;12:19416. doi: 10.1038/s41598-022-23996-y (PMC9653406; doi:10.1038/s41598-022-23996-y)
Supplement: Supplementary file 1 — Supplementary Information. [file 41598_2022_23996_MOESM1_ESM.pdf]

SARS-CoV-2 detection by targeting four loci of viral genome using graphene oxide and gold nanoparticle DNA biosensors

Arman Amani Babadi[1,2,+], Shahrooz Rahmati[3,4,5,6,+,*], Rafieh Fakhlaei[7,+], Reza Heidari[8,+], Saeid Baradaran[9], Mostafa Akbariqomi[10], Shuang Wang[1,*], Gholamreza Tavoosidana[2,*], William Doherty[4], Kostya (Ken) Ostrikov[3,4,5,6]

[1] School of Energy and Power Engineering, Jiangsu University, Jiangsu, 212013, China
[2] Department of Molecular Medicine, School of Advanced Technologies in Medicine, Tehran University of Medical Sciences, Tehran, 55469-14177, Iran
[3] School of Chemistry and Physics, Queensland University of Technology (QUT), Brisbane 4000, Australia
[4] Centre for Agriculture and the Bioeconomy, Queensland University of Technology (QUT), Brisbane 4000, Australia
[5] Centre for Materials Science, Queensland University of Technology (QUT), 2 George Street, Brisbane 4000, Australia
[6] Centre for Biomedical Technologies, Queensland University of Technology (QUT), 2 George Street, Brisbane 4000, Australia
[7] Food Safety and Food Integrity (FOSFI), Institute of Tropical Agriculture and Food Security, Universiti Putra Malaysia, Serdang 43400, Selangor, Malaysia
[8] Research Center for Cancer Screening and Epidemiology, AJA University of Medical Sciences, Tehran, 14117-18541, Iran
[9] New Technologies Research Center, Amirkabir University of Technology, Tehran, 15916-34311, Iran
[10] Applied Microbiology Research Center, Systems Biology and Poisonings Institute, Baqiyatallah University of Medical Sciences, Tehran, 14359-16471, Iran

[*]Corresponding author Email: shahrooz.rahmati@hdr.qut.edu.au; Alexjuven@ujs.edu.cn; g-Tavoosi@tums.ac.ir
[+]These authors contributed equally to this work

Table S1 The BLAST result of SCVP1

No	Accession	Identity %	Starting Target Position	No	Accession	Identity %	Starting Target Position	No	Accession	Identity %	Starting Target Position
1	MT890669.1	100	29155	41	MT890320.1	100	29149	81	MT890280.1	100	29152
2	MT890462.1	100	29159	42	MT890319.1	100	29147	82	MT890279.1	100	29110
3	MT890358.1	100	29158	43	MT890318.1	100	29123	83	MT890278.1	100	29120
4	MT890357.1	100	29157	44	MT890317.1	100	29121	84	MT890277.1	100	29151
5	MT890356.1	100	29157	45	MT890316.1	100	29121	85	MT890276.1	100	28972
6	MT890355.1	100	29155	46	MT890315.1	100	29121	86	MT890275.1	100	29121
7	MT890354.1	100	29142	47	MT890314.1	100	29155	87	MT890274.1	100	29121
8	MT890353.1	100	29158	48	MT890313.1	100	29121	88	MT890273.1	100	29121
9	MT890352.1	100	29157	49	MT890312.1	100	29121	89	MT890272.1	100	29120
10	MT890351.1	100	29121	50	MT890311.1	100	29156	90	MT890249.1	100	29121
11	MT890350.1	100	29121	51	MT890310.1	100	29150	91	MT890248.1	100	29120
12	MT890349.1	100	29155	52	MT890309.1	100	29123	92	MT890247.1	100	29121
13	MT890348.1	100	29121	53	MT890308.1	100	29147	93	MT890246.1	100	29120
14	MT890347.1	100	29158	54	MT890307.1	100	29120	94	MT890245.1	100	29121
15	MT890346.1	100	29121	55	MT890306.1	100	28829	95	MT890244.1	100	29120
16	MT890345.1	100	29121	56	MT890305.1	100	28829	96	MT890243.1	100	29120
17	MT890344.1	100	29148	57	MT890304.1	100	29121	97	MT890242.1	100	29120
18	MT890343.1	100	29121	58	MT890303.1	100	28834	98	MT890241.1	100	29120
19	MT890342.1	100	29121	59	MT890302.1	100	29121	99	MT890240.1	100	29120
20	MT890341.1	100	29149	60	MT890301.1	100	29156	100	MT890239.1	100	29120
21	MT890340.1	100	29129	61	MT890300.1	100	28830				
22	MT890339.1	100	29121	62	MT890299.1	100	29123				
23	MT890338.1	100	29155	63	MT890298.1	100	28830				
24	MT890337.1	100	29149	64	MT890297.1	100	28831				
25	MT890336.1	100	29151	65	MT890296.1	100	29158				
26	MT890335.1	100	29121	66	MT890295.1	100	29157				
27	MT890334.1	100	29148	67	MT890294.1	100	29157				
28	MT890333.1	100	29152	68	MT890293.1	100	29147				
29	MT890332.1	100	28829	69	MT890292.1	100	29157				
30	MT890331.1	100	28829	70	MT890291.1	100	29121				
31	MT890330.1	100	29144	71	MT890290.1	100	29149				
32	MT890329.1	100	29033	72	MT890289.1	100	29147				
33	MT890328.1	100	28829	73	MT890288.1	100	29033				
34	MT890327.1	100	29121	74	MT890287.1	100	28838				
35	MT890326.1	100	28829	75	MT890286.1	100	29150				
36	MT890325.1	100	29121	76	MT890285.1	100	29158				
37	MT890324.1	100	28831	77	MT890284.1	100	29121				
38	MT890323.1	100	28829	78	MT890283.1	100	29120				
39	MT890322.1	100	29121	79	MT890282.1	100	29147				
40	MT890321.1	100	29156	80	MT890281.1	100	29111				

Table S2 The BLAST result of SCVP2

No	Accession	Identity %	Starting Target Position	No	Accession	Identity %	Starting Target Position	No	Accession	Identity %	Starting Target Position
1	MT890669.1	100	28712	41	MT890320.1	100	28706	81	MT890280.1	100	28709
2	MT890462.1	100	28716	42	MT890319.1	100	28704	82	MT890279.1	100	28667
3	MT890358.1	100	28715	43	MT890318.1	100	28680	83	MT890278.1	100	28677
4	MT890357.1	100	28714	44	MT890317.1	100	28678	84	MT890277.1	100	28708
5	MT890356.1	100	28714	45	MT890316.1	100	28678	85	MT890276.1	100	28529
6	MT890355.1	100	28712	46	MT890315.1	100	28678	86	MT890275.1	100	28678
7	MT890354.1	100	28699	47	MT890314.1	100	28712	87	MT890274.1	100	28678
8	MT890353.1	100	28715	48	MT890313.1	100	28678	88	MT890273.1	100	28678
9	MT890352.1	100	28714	49	MT890312.1	100	28678	89	MT890272.1	100	28677
10	MT890351.1	100	28678	50	MT890311.1	100	28713	90	MT890249.1	100	28678
11	MT890350.1	100	28678	51	MT890310.1	100	28707	91	MT890248.1	100	28677
12	MT890349.1	100	28712	52	MT890309.1	100	28680	92	MT890247.1	100	28678
13	MT890348.1	100	28678	53	MT890308.1	100	28704	93	MT890246.1	100	28677
14	MT890347.1	100	28715	54	MT890307.1	100	28677	94	MT890245.1	100	28678
15	MT890346.1	100	28678	55	MT890306.1	100	28386	95	MT890244.1	100	28677
16	MT890345.1	100	28678	56	MT890305.1	100	28386	96	MT890243.1	100	28677
17	MT890344.1	100	28705	57	MT890304.1	100	28678	97	MT890242.1	100	28677
18	MT890343.1	100	28678	58	MT890303.1	100	28391	98	MT890241.1	100	28677
19	MT890342.1	100	28678	59	MT890302.1	100	28678	99	MT890240.1	100	28677
20	MT890341.1	100	28706	60	MT890301.1	100	28713	100	MT890239.1	100	28677
21	MT890340.1	100	28686	61	MT890300.1	100	28387				
22	MT890339.1	100	28678	62	MT890299.1	100	28680				
23	MT890338.1	100	28712	63	MT890298.1	100	28387				
24	MT890337.1	100	28706	64	MT890297.1	100	28388				
25	MT890336.1	100	28708	65	MT890296.1	100	28715				
26	MT890335.1	100	28678	66	MT890295.1	100	28714				
27	MT890334.1	100	28705	67	MT890294.1	100	28714				
28	MT890333.1	100	28709	68	MT890293.1	100	28704				
29	MT890332.1	100	28386	69	MT890292.1	100	28714				
30	MT890331.1	100	28386	70	MT890291.1	100	28678				
31	MT890330.1	100	28701	71	MT890290.1	100	28706				
32	MT890329.1	100	28590	72	MT890289.1	100	28704				
33	MT890328.1	100	28386	73	MT890288.1	100	28590				
34	MT890327.1	100	28678	74	MT890287.1	100	28395				
35	MT890326.1	100	28386	75	MT890286.1	100	28707				
36	MT890325.1	100	28678	76	MT890285.1	100	28715				
37	MT890324.1	100	28388	77	MT890284.1	100	28678				
38	MT890323.1	100	28386	78	MT890283.1	100	28677				
39	MT890322.1	100	28678	79	MT890282.1	100	28704				
40	MT890321.1	100	28713	80	MT890281.1	100	28668				

Table S3 The BLAST result of SCVP3

No	Accession	Identity %	Starting Target Position	No	Accession	Identity %	Starting Target Position	No	Accession	Identity %	Starting Target Position
1	MT890669.1	100	26328	41	MT890320.1	100	26322	81	MT890281.1	100	26325
2	MT890462.1	100	26332	42	MT890319.1	100	26320	82	MT890280.1	100	26283
3	MT890358.1	100	26331	43	MT890318.1	100	26296	83	MT890279.1	100	26293
4	MT890357.1	100	26330	44	MT890317.1	100	26294	84	MT890278.1	100	26324
5	MT890356.1	100	26330	45	MT890316.1	100	26294	85	MT890277.1	100	26145
6	MT890355.1	100	26328	46	MT890315.1	100	26294	86	MT890276.1	100	26294
7	MT890354.1	100	26315	47	MT890314.1	100	26328	87	MT890275.1	100	26294
8	MT890353.1	100	26331	48	MT890313.1	100	26294	88	MT890274.1	100	26294
9	MT890352.1	100	26330	49	MT890312.1	100	26294	89	MT890273.1	100	26293
10	MT890351.1	100	26294	50	MT890311.1	100	26329	90	MT890272.1	100	26294
11	MT890350.1	100	26294	51	MT890310.1	100	26323	91	MT890249.1	100	26293
12	MT890349.1	100	26328	52	MT890309.1	100	26296	92	MT890248.1	100	26294
13	MT890348.1	100	26294	53	MT890308.1	100	26320	93	MT890247.1	100	26293
14	MT890347.1	100	26331	54	MT890307.1	100	26293	94	MT890246.1	100	26294
15	MT890346.1	100	26294	55	MT890306.1	100	26002	95	MT890245.1	100	26293
16	MT890345.1	100	26294	56	MT890305.1	100	26002	96	MT890244.1	100	26293
17	MT890344.1	100	26321	57	MT890304.1	100	26294	97	MT890243.1	100	26293
18	MT890343.1	100	26294	58	MT890303.1	100	26007	98	MT890242.1	100	26293
19	MT890342.1	100	26294	59	MT890302.1	100	26294	99	MT890241.1	100	26293
20	MT890341.1	100	26322	60	MT890301.1	100	26329	100	MT890240.1	100	26293
21	MT890340.1	100	26302	61	MT890300.1	100	26003				
22	MT890339.1	100	26294	62	MT890299.1	100	26296				
23	MT890338.1	100	26328	63	MT890298.1	100	26003				
24	MT890337.1	100	26322	64	MT890297.1	100	26004				
25	MT890336.1	100	26324	65	MT890296.1	100	26331				
26	MT890335.1	100	26294	66	MT890295.1	100	26330				
27	MT890334.1	100	26321	67	MT890294.1	100	26330				
28	MT890333.1	100	26325	68	MT890293.1	100	26320				
29	MT890332.1	100	26002	69	MT890292.1	100	26330				
30	MT890331.1	100	26002	70	MT890291.1	100	26294				
31	MT890330.1	100	26317	71	MT890290.1	100	26322				
32	MT890329.1	100	26206	72	MT890289.1	100	26320				
33	MT890328.1	100	26002	73	MT890288.1	100	26206				
34	MT890327.1	100	26294	74	MT890287.1	100	26011				
35	MT890326.1	100	26002	75	MT890286.1	100	26323				
36	MT890325.1	100	26294	76	MT890285.1	100	26331				
37	MT890324.1	100	26004	77	MT890284.1	100	26294				
38	MT890323.1	100	26002	78	MT890283.1	100	26293				
39	MT890322.1	100	26294	79	MT890282.1	100	26320				
40	MT890321.1	100	26329	80	MT890320.1	100	26284				

Table S4 The BLAST result of SCVP4

No	Accession	Identity %	Starting Target Position	No	Accession	Identity %	Starting Target Position	No	Accession	Identity %	Starting Target Position
1	MT890669.1	100	15466	41	MT890320.1	100	15460	81	MT890280.1	100	15469
2	MT890462.1	100	15470	42	MT890319.1	100	15458	82	MT890279.1	100	15421
3	MT890358.1	100	15469	43	MT890318.1	100	15434	83	MT890278.1	100	15431
4	MT890357.1	100	15468	44	MT890317.1	100	15432	84	MT890277.1	100	15462
5	MT890356.1	100	15468	45	MT890316.1	100	15432	85	MT890276.1	100	15283
6	MT890355.1	100	15466	46	MT890315.1	100	15432	86	MT890275.1	100	15432
7	MT890354.1	100	15453	47	MT890314.1	100	15466	87	MT890274.1	100	15432
8	MT890353.1	100	15469	48	MT890313.1	100	15432	88	MT890273.1	100	15432
9	MT890352.1	100	15468	49	MT890312.1	100	15432	89	MT890272.1	100	15431
10	MT890351.1	100	15432	50	MT890311.1	100	15467	90	MT890249.1	100	15432
11	MT890350.1	100	15432	51	MT890310.1	100	15461	91	MT890248.1	100	15431
12	MT890349.1	100	15466	52	MT890309.1	100	15434	92	MT890247.1	100	15432
13	MT890348.1	100	15432	53	MT890308.1	100	15458	93	MT890246.1	100	15431
14	MT890347.1	100	15469	54	MT890307.1	100	15431	94	MT890245.1	100	15432
15	MT890346.1	100	15432	55	MT890306.1	100	15140	95	MT890244.1	100	15431
16	MT890345.1	100	15432	56	MT890305.1	100	15140	96	MT890243.1	100	15431
17	MT890344.1	100	15459	57	MT890304.1	100	15432	97	MT890242.1	100	15431
18	MT890343.1	100	15432	58	MT890303.1	100	15145	98	MT890241.1	100	15431
19	MT890342.1	100	15432	59	MT890302.1	100	15432	99	MT890240.1	100	15431
20	MT890341.1	100	15460	60	MT890301.1	100	15467	100	MT890239.1	100	15431
21	MT890340.1	100	15440	61	MT890300.1	100	15141				
22	MT890339.1	100	15432	62	MT890299.1	100	15434				
23	MT890338.1	100	15466	63	MT890298.1	100	15141				
24	MT890337.1	100	15460	64	MT890297.1	100	15142				
25	MT890336.1	100	15462	65	MT890296.1	100	15469				
26	MT890335.1	100	15432	66	MT890295.1	100	15468				
27	MT890334.1	100	15459	67	MT890294.1	100	15468				
28	MT890333.1	100	15463	68	MT890293.1	100	15458				
29	MT890332.1	100	15140	69	MT890292.1	100	15468				
30	MT890331.1	100	15140	70	MT890291.1	100	15432				
31	MT890330.1	100	15455	71	MT890290.1	100	15460				
32	MT890329.1	100	15344	72	MT890289.1	100	15458				
33	MT890328.1	100	15140	73	MT890288.1	100	15344				
34	MT890327.1	100	15432	74	MT890287.1	100	15149				
35	MT890326.1	100	15140	75	MT890286.1	100	15461				
36	MT890325.1	100	15432	76	MT890285.1	100	15469				
37	MT890324.1	100	15142	77	MT890284.1	100	15432				
38	MT890323.1	100	15140	78	MT890283.1	100	15431				
39	MT890322.1	100	15432	79	MT890282.1	100	15458				
40	MT890321.1	100	15467	80	MT890281.1	100	15422				

Table S5 Physical characteristics of SCVPs

SCVP	Molecular Weight	Extinction Coefficient	Length (bp)	Tm (°C)	GC%
1	6147.1	195.4	20	58.9	40.0
2	6228.1	168.1	20	65.9	50
3	7796.1	252.9	25	72.0	56
4	7609.0	227.9	25	72.6	56

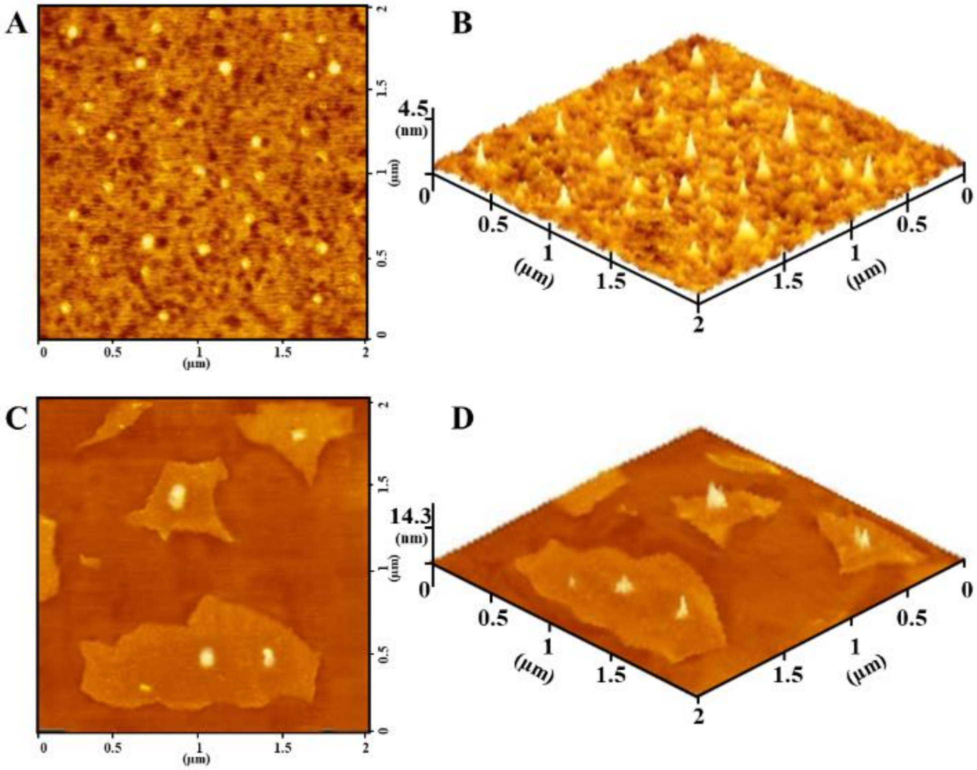

Figure S1 AFM topography 2D and 3D images of SCVPs capped Au-NPs (a-b) and SCVP4 capped GO/Au-NPs (c, d) adsorbed on mica surface.
